# Hospital Meetings

**Published:** 1903-04-11

**Authors:** 


### HOSPITAL ADMINISTRATION.

### CONSTRUCTION AND ECONOMICS.

### Hospital Meetings.

### UNIVERSITY COLLEGE HOSPITAL.

Lord Monkswell, the treasurer, presided at the annual meeting of the North London or University College Hospital on the 19th instant.

In moving the adoption of the report he said they would see with great satisfaction that the Prince of Wales had accepted the position of Vice-Patron, and also that steady progress was being made with the new building, but they would not be so glad that the committee had not been able to proceed with the erection of the proposed private nursing home, for which funds were not forthcoming. They had, however, bought the freehold of five houses in University Street and two in Chenies Mews for the purpose, and hoped friends would come forward and help them build this necessary adjunct to their work. During the year they had installed Finsen light and radiographic departments and these were now at work. The Ladies' Association, he was glad to point out, had been doing most useful work. As to the contributions during the past year there was little to be said, the comparison being about the same as in 1901. The Sunday Fund had given them £1,303, and the Saturday Fund £364. £3,500 had been received from King Edward's Fund for 1902, £2,500 of this being awarded for the payment of tradesmen's bills. The People's Contribution Fund had sent them £400. They had been exceedingly fortunate in the amount of their legacies, which totalled £35,000. If that sum could be depended on each year they would have very little cause for anxiety, but, unfortunately, legacies fluctuated very considerably. They would have liked to invest the whole of that sum, but a large amount had had to be taken from it to meet current tradesmen's bills, which had made a considerable inroad into it. They had also had to meet expenditure in connection with the new building—temporary adaptations, furnishing and fitting the new wards as they became available, and so forth. The response to their customary Christmas appeal had not been so large as usual, and he thought it possible that people were giving their money to King Edward's and other central funds instead. Their expenditure had decreased by the substantial sum of £1,443, and this showed that they had been on the alert with regard to cutting down their expenses so far as that was compatible with efficiency. They greatly regretted to have to record the death of Mrs. Nathaniel Montefiore, who had always been a great friend to the hospital and had left them £2,000 to be invested. In conclusion, he desired to draw the attention of the public to the financial condition of the hospital. The better accommodation provided in the new building was of immense advantage to the patients, but it was much more expensive to maintain. There was much more room and many costly appliances, and every new wing opened caused further expense. When the new building was completed and all the 300 beds open they hoped the cost per bed would be materially diminished. Despite rigid economy their annual expenditure was nearly £24,000, and toward this their reliable income was only £8,000, the difference having to be made up by subscriptions and donations for which they earnestly pleaded.

Mr. Lucas, the chairman of the committee, stated that there was a very good prospect of the whole building being finished by the end of 1904. They were, in fact, told that this would be so by their builders. Sir Blundell Maple had promised to pay £120,000, but the expenditure by him had already far exceeded that sum. Whether they would be able to open the full number of 300 beds must depend on the public and the amount of subscriptions they received; certainly if they got no more support than at present they would not be able to. It would take £30,000 a year, which would mean that they would have to raise at least £22,000. Not long since they had been on the point of closing some of their beds. He trusted, therefore, that the benefits the hospital was conferring on the public, and especially on the public of that district, would be more adequately recognised.

The adoption of the report was agreed to, and votes of thanks brought the proceedings to a close.

### NORTH-EASTERN HOSPITAL FOR CHILDREN.

A special meeting was held at the Mansion House on March 23rd in aid of the Building Fund of this hospital. The chair was taken by the Lord Mayor, and with him on the platform were the Duchess of Somerset, the Lady Mayoress, Lord and Lady Frederick Fitzroy, Alderman Sir William P. Treloar, and other members of the committee.

The Lord Mayor, in his introductory speech, stated that he had taken a great interest in this hospital for some years. His lordship alluded to the very practical way in which the juvenile readers of the "Little Folks" magazine had assisted the hospital by raising £1,200 out of £2,000 necessary for the establishment of a "Little Folks" ward of six cots, and went on to demonstrate how great was the need of assistance for the Building Fund, which originally had been estimated at £30,000, but now proved to be £32,000. In the same way the Nurses' Home would cost £25,000 instead of £20,000, as had been at first hoped; so that altogether the committee stood in need of the very large sum of £32,000 in order to carry out absolutely necessary improvements.

Dr. C. A. James (Chairman of the Hackney Bazaar of December 1902) testified to the great interest taken by the inhabitants of Hackney in the North-Eastern Hospital, and the strenuous efforts they had made in its behalf at the recent bazaar, instancing the fact that 30,000 children in the Board schools of that neighbourhood had contributed £300. A cheque for £1,684, which represented the net proceeds of the bazaar, was then presented to the Lord Mayor by the treasurer.

The Duchess of Somerset then moved the following resolution:—That this meeting is of opinion that the scheme of enlargement of the North-Eastern Hospital ought in the public interest to be carried out in its entirety without delay, and that this meeting desires to draw general attention to the urgency of the need for further contributions to place the committee of the hospital in a position to give effect to their plans." The Duchess said that a meeting like the present did much good by making people come together and work for a definite object, and it was much to be hoped that this appeal would meet with the support it deserved. The children of to-day would be the nation of to-morrow, and by assisting these children they would be strengthening the hands of the nation of the future. People who were unable to give much money could at any rate give work. It was a very mistaken idea to think that giving money was the only way of rendering assistance; they could try and interest their friends in the work of the hospital. Enormous sums of money were constantly raised in London, and her Grace said she earnestly hoped the result of the meeting would be that the entire sum required would be raised. It was three years since Lady Frederick Fitzroy had enlisted her sympathies in the North-Eastern Hospital. As regards the accommodation for the nurses, only two nurses were able to reside at the hospital; the others had to take a tram some little distance to the lodging-house provided for them. This was not at all a proper state of things. The Duchess ended by a fervent appeal to all present to contribute liberally to this fund and to put the hospital on a proper footing.

Alderman Sir William Treloar, who seconded the resolution, said that if the required money were forthcoming they trusted they would have the building ready for occupation by next June. The enlargements had been pressed on the committee by the Visiting Committee of King Edward's Hospital Fund.

The Rev. Dr. Adler, who also spoke in support of the resolution, said that this might be termed hospital year, for there was so much going on in connection with the extension, rebuilding, and removal of the larger hospitals; but that the needs of the smaller hospitals ought not to be passed over, and they as well as the larger ones should be rendered worthy of support, both by reason of the efficiency of their administration and the sufficiency of their accommodation. The efficiency of administration they already possessed, but the sufficiency of accommodation was urgently required.

A vote of thanks was then passed to the Lord Mayor for presiding.

### HOSPITAL FOR WOMEN, SOHO SQUARE.

A special meeting on behalf of this hospital was held at the Mansion House on March 27th, at which the Lord Mayor presided. H.R.H. Princess Christian (one of the patronesses) attended the meeting.

The Lord Mayor, after thanking H.R.H. Princess Christian for her attendance at the meeting and for the interest she had shown at all times in the hospital, said that the question of the removal of hospitals was one that was attracting much attention at the present time, but the first thing to be proved was whether there existed a need for a hospital in a certain neighbourhood, and if there did, then no matter how tempting the financial benefits resultant on removal might appear, it was the first duty of the hospital to supply the needs of that district. The Lord Mayor went on to say that he hoped that the seed sown at the meeting would result in a large crop of donations.

Mr. V. A. Williamson, Chairman of the Board of Management, said that of late years many of the original subscribers to the hospital had passed away and their places had not been filled, and it was only owing to the legacies received that the hospital had been able to continue its work. The nurses' quarters had been put into good order some 14 years ago and there was now no fault to be found with them, but much required to be done in other parts of the building. Mr. Williamson commented on the rapidity of the advances made in these days; a hospital that was rendered completely up-to-date to-day would in 10 years time be obsolete. Two years ago they had been obliged to close 25 beds out of 60, but the grant they had received from King Edward's Hospital Fund had enabled them to re-open them again, but even with that grant they needed an additional £200 a year in annual subscriptions. As to the question of removal, Mr. Williamson said he had lately attended a lecture given by Sir Henry Burdett, and one of the principles therein laid down was that the first essential for hospitals was accessibility. Their out-patient department was a very large one, the attendances in 1901 amounting to 18,000. The great bulk of the out-patients were Londoners, chiefly from the East End, and it was the speaker's contention that the Soho Hospital was situated in as accessible a spot as any in London, and that any removal to the suburbs would militate against the success of the out-patient department. Mr. Williamson scouted the idea that the air of Soho could not be considered healthy, and advocated the building of a convalescent home, or country branch, either in the neighbourhood of London or in the country.

Sir Edmund Hay Currie stated his opinion that the cheapest and best thing to do with the hospital was to pull it down and rebuild it entirely. If they patched it up now the result would be that in a few years time they would have to go through exactly the same thing again. The hospital was known all over the country for the excellent work it performed, and it ought to be put on such a footing that it would occasion no further anxiety. The support of the three great Hospital Funds—King Edward's, the Sunday and Saturday Funds—was the hall mark of a well-conducted hospital. The Sunday Fund had been unable to give the Women's Hospital the full grant, not because it was not deserved, but because that Fund had to consider the question of the cost of each patient. The hospital was not in a position to be worked economically, it was an expensive one, and cost more than it should do. The only way to lessen the expenses was to have as many labour-saving appliances as possible. The report of King Edward's Fund also called attention to the cost of each individual patient. The hospital would never be a success if it were patched up. Sir Edmund Currie said he did not press for removal, and thought the idea of a country branch was an excellent one, but the committee should reconsider the question of the sum they should appeal for, and instead of £10,000 they should ask for £25,000, which would be the cost of a new hospital; this sum, he was sure, they would have no difficulty in obtaining. The hospital, though a comfortable one, was never intended for a hospital.

The Hon. Alban Gibbs, M.P., and Lady Kathleen Bligh also supported the claims of the hospital, and appealed for the aid of the wealthy in the City and throughout the country.

### ROYAL EYE HOSPITAL.

The annual meeting of this hospital was held on March 18th, when the necessary business of receiving the report and electing the new council was transacted. If, however, as the result of a special effort the governors were induced to attend the annual meeting, the council would almost certainly find public interest in the hospital quickened, and arising therefrom, an increase in the subscriptions, and a corresponding extension of the work of the hospital. Such an effort could hardly be considered as an advertisement, and might go far towards removing the inadequacy of the financial support which the council had again to lament.

The chair was taken by Mr. W. Moresby-Chinnery, and the meeting was addressed by the Senior Surgeon and Vice-President, Professor Malcolm McHardy.

The report recorded, said the Professor, the increasing demands upon the resources of the hospital and the far from corresponding augmentation of their funds. It showed how nearly 20,000 poor sufferers had been assisted with an expenditure of only £3,319. The ministrations of the hospital were limited by the Council's exceptional practice of keeping out of debt; but such morality in hospital finances did not meet with the encouragement it deserved. The street noises and incessant rattle of the traffic were a great drawback, but the substitution of wood paving for granite had given the hospital much relief. The work of the hospital had trebled in the last 20 years, and in the Professor's opinion the need of such work must increase as long as compulsory education was in force for the young poor. The out-patient department was now worked with a double shift, being open at 9 every morning and again from 2 to 3 in the afternoon.

The Chairman commented on the difficulty experienced by the hospital in obtaining money, and said that it was doing work which was not thoroughly known to the philanthropic public, and it was their duty to make it better known. He believed that they would then obtain an income which would enable them to supply the needs of the hospital.

Votes of thanks to the honorary medical officers and staff having been passed, the meeting adjourned.

### POPLAR HOSPITAL FOR ACCIDENTS.

Mr. J. G. Broodbank, Vice-chairman, presided over the annual meeting of the Poplar Hospital for Accidents on March 24th. The Chairman in his opening remarks, said that the year 1902 had been a thoroughly satisfactory one. Their income had sufficed for all their needs, and they had over £600 to the good, besides having been able to add to their reserve funds the sum of £2,000. This state of things at the end of what had certainly been a bad year for hospitals was most encouraging. The Chairman was able to assure the meeting that no further capital expenditure was contemplated. They had paid for all the building they had undertaken, and there were no further claims to meet on that account. The Chairman, in conclusion, said Mr. Sydney Holland had particularly requested him to acknowledge how great a debt the committee and the hospital owed to its most efficient staff of matrons, sisters, and nurses. The adoption of the report having been moved, it was seconded by Colonel J. L. Du Plat Taylor, who said he felt inclined to lay claim to some small share of credit for the present satisfactory position of the hospital, since he had been the first to induce Mr. Sydney Holland to take an interest in hospital work. He had also been the means of introducing Colonel Feneran to the work of the hospital, so that he felt that the hospital was in a slight degree indebted to him. The necessary resolutions having been passed, the general meeting was resolved into a committee meeting.

### BRITISH LYING-IN HOSPITAL.

Great progress has been made at this hospital with the nurses' home. Mr. Charles E. Farmer alluded to the subject on Tuesday (March 24th), when presiding over the annual meeting of the hospital. The home, he announced, was approaching completion, and H.R.H. the Princess of Wales had graciously intimated that she would perform the opening ceremony on June 8th. The completion of the home would set free two additional wards, which, in view of the increasing work of the hospital, were badly needed. The number of in-patients was more than double the number treated five years ago. The figures were, however, somewhat lower than in 1901, owing chiefly to the dislike of patients to enter the hospital during the time when visitors were excluded on account of the small-pox epidemic. On the other hand, the out-patients had more than correspondingly increased. There were no maternal deaths among the out-patients, but four had occurred in the hospital. These, however, were cases complicated by disease; two patients were in an advanced stage of consumption, the other two were suffering from grave cardiac disease, and from hæmorrhagic small-pox. Three were in a dying condition when admitted. In-patients were applying for admission in very large numbers; and recently seven children were born in the hospital in one day; from this it might be judged that the work was growing. This was so evident to the Board that they had appointed a second assistant matron, who would be responsible for the out-patients' department; this would relieve Miss Knott and her indoor assistant somewhat.

The finances were short by £50. This deficiency would probably increase, since the rent of the house in Betterton Street—£80 a year—was relinquished in order to build the Nurses' Home on the site; the home itself would also cause additional expense. Against this, of course, must be set the fees received from a larger number of nurses and pupils.

The Council of King Edward's Fund, the Chairman continued, had entirely withdrawn their comment which appeared in the daily Press, that they considered the hospital extravagant with regard to the nurses' home. On fuller inquiry, they had altered their opinion, and had decided to make no reference to the matter in their report. He hoped they might increase their grant next year.

The report and accounts were adopted unanimously, and on the motion of Lord Kinnaird, Mr. Frederick Cox, who retires after 30 years' work for the hospital, was elected vice-President.

### ROYAL DENTAL HOSPITAL.

Lord Kinnaird took the chair at the annual general meeting of governors, held on March 31st, and said that the committee of management had again to report a large increase in the number of cases treated during 1902, the total amounting to 85,284, being 15,244 in excess of the total for 1901. The Chairman remarked that he himself received far more applications for letters of admission into the Royal Dental Hospital than into any other, and he hoped the public would furnish them with the means to carry on the work that was so much appreciated. The receipts for the past year showed an increase under every head except that of legacies. The amounts received from the Hospital Sunday and Saturday Funds were also greater, and the sum of £300 had been contributed by King Edward's Hospital Fund towards the liquidation of the debt on the hospital. A special appeal had been issued to pay off the debt on the operating chairs, which cost £15 15s. each and the sum of £889 15s. had been received for this purpose, 32 chairs still remaining unpaid for. Lord Kinnaird then went on to mention an important decision which the committee had recently arrived at, namely the surrender of the license of the public house immediately adjoining the hospital. The committee had for some time past been convinced that the presence of this public house was most undesirable, but they had naturally been reluctant to relinquish so valuable an asset. The Chairman then stated that the public house had been closed that day and proceeded to destroy the license, remarking that he trusted the subscribers would not allow the hospital to be the losers by this transaction.

### WEST LONDON HOSPITAL.

At the annual meeting of the Governors of this hospital held on April 2, Mr. J. H. Lewis, the Chairman, alluded to the great loss the hospital had sustained during the past year through the death of Mr. William Bird, Chairman of the Board. Lord Glenesk, said Mr. Lewis, had consented to fill this post. Dealing with the report for the year 1902, the Chairman said it might be considered very satisfactory, their income being now in excess of the expenditure, and the heavy debt incurred by the hospital in past years having been materially reduced, thanks to large legacies received and the substantial assistance afforded by King Edward's Fund. The Board contemplated adding a new post-mortem department, a mortuary, and a new pathological department, at a cost of about £3,500. This last was urgently needed for the prompt and rapid diagnosis of disease. Improvements were also needed in very many directions, especially in the casualty department, which was quite inadequate, and required enlarging and altering; the same remark applying to the nurses' quarters. It had also been pressed upon the Board by the surgeons that the theatre accommodation of the hospital was insufficient. All these improvements were a matter of money. The Board were quite ready to remedy these defects if only they could obtain the necessary funds. They were about to establish a ladies' association, which the board hoped would add to the efficiency of the hospital and be of great assistance in obtaining the funds they so urgently needed.

Mr. Arthur Watson, who was the next speaker, said that although the receipts for the past year exceeded the expenditure, this had only been accomplished by treating as ordinary revenue the legacies which in more prosperous institutions would have been invested and treated as capital. It was much to be regretted that these legacies had been so treated. Had it not been for the large grants they had received from King Edward's Fund the hospital would have been out of pocket at the close of last year. This was not a dead and alive hospital, it was very much alive, and always built in advance of the funds and endeavoured in every way to keep up with the demands of the present day. The hospital was managed in the most economical way possible, as was proved by the fact that the cost of each patient amounted to only 25s. per week.

The chief event recorded in the report for 1902 was the completion and opening of the Annie Zunz Ward, which had added 26 beds to the hospital accommodation and had brought the total number of available beds in the general wards up to 150.

### CITY OF LONDON HOSPITAL FOR DISEASES OF THE CHEST.

Sir Edward Sassoon, the treasurer, presided at the fifty-fifth annual meeting of the City of London Hospital for Diseases of the Chest, Victoria Park.

In moving the adoption of the report and accounts the Chairman said he thought the latter showed that all the outgoings and incomings during the year had been admirably maintained. Donations were £500 better than last year from the Hospital Sunday Fund they received £300 more, while the amount allocated to them from King Edward's Fund—£1,500—was the same also; but he must add that the idea of the committee to establish a nursing home, which had become a matter of vital necessity, had met with the approval of that body, and they had sent them a special award of £750 towards it. With regard to their disbursements, it was not unsatisfactory, he thought, that considering the increase in the work their expenses had not increased, there had, in fact, been an actual decrease in some departments. They might, he considered, without impropriety take credit for having maintained a high standard, adopting the most modern methods and improved appliances in connection with the work of the hospital. He could not but think that those who prospered and throve in the City, and whose human instruments of production and distribution often fell out by the way a prey to that most fell disease, to combat which that hospital was maintained, might render them more assistance than they did. One reason given for this failure sometimes was that, owing to the action of King Edward's Fund, the money went into that channel. He could not help thinking, however, that it was due to inadequate and incomplete knowledge of the work done. This was certainly no fault of their indefatigable secretary, who did all that was possible to make their needs known. It might be interesting to know how many large employers of workmen abstained from subscribing, and he commended the idea to the secretary, in his leisure moments, to compile statistics showing this. But if their friends would make known their work, and their need, and in this way it would be of the greatest service. As to the Ladies' Association, it was impossible to detail the amount of good done by them in a quiet and unostentatious fashion that made it the more gracious. They were under a debt of gratitude to Sir Charles Wyndham for the loan of his theatre, which resulted in an addition of £800 to their funds. The question of a nurses' home was not making the progress they could desire, but this was not so much a matter of surprise as of regret. They hoped, however, next year to achieve far greater progress. He concluded his remarks with a warm tribute to the services of the secretary and matron.

On the motion to re-elect the members of the Committee of Management one of the working-men governors present raised a question as to the attendances of the House Subcommittee, stating that when patients were discharged they sometimes saw only two members and the secretary. He was assured, however, that two formed a quorum, which was found to be quite adequate for the purpose, and that a patient, having any representation to make, had an opportunity of explaining the matter to the secretary beforehand.

Sir Edmund Currie rose to move that the thanks of the Governors be given to the Committee of Management. Judged by the results obtained they were extremely economical. The cost per patient was very low, 30s. per week as against £2 6s. elsewhere, and at the same time the work was most efficiently carried out. This could only be accomplished by a committee of management carefully watching every detail. He did not want to draw invidious comparisons, but it was clear that of the four chest hospitals this was the cheapest, and there was no doubt a very great difference was made by having a house committee of two, with the assistance of so excellent a secretary, sitting week by week. He was sorry they did not seem very sanguine about the nurses' home. He had recently visited the hospital, and after noticing the very admirable way the open-air treatment was being carried on, he had no hesitation in saying that he thought it was desirable its scope should be increased, as the erection of a nurses' home would enable them to increase it. He gathered from the secretary that great need was felt for additional galleries. He had no hesitation in saying this was quite one of the best hospitals, and he had been struck with the tone of good honest work. He remembered when they had only 16 beds open; now they had 120 full out of 140. If the committee were not doing good work he would not have been able to speak as he had done, and he had therefore great pleasure in moving this vote of thanks to them.

Dr. Heron, in moving a vote of thanks to the Chairman, referred to the enormous improvement in the hospital's condition since he became connected with it. Six years ago he said they owed £6,000 to their bankers, and had closed their out-patient department because they had no money with which to carry it on; in fact, the end seemed very near. Then they got new blood into the management, and Sir Edward Sassoon became treasurer. Within eighteen months they had paid off £3,000 of that debt, had raised the income from a paltry sum to £11,000 a year, and in various ways had put a new complexion on things. What was practically a hospital in its dying agony had been put on its legs and made a feature in the hospital life of London, ranking with any institution of the kind either in or out of England.

Therefore their thanks were not a matter of form, since he had played no inconsiderable part in dragging them from the mire.

### LONDON TEMPERANCE HOSPITAL.

The London Temperance Hospital makes a great feature of their annual meeting day. The wards are open to inspection between the hours of 4 and 5 P.M. and 6 and 7 P.M. The Governors' meeting takes place at 5 P.M., at which all the ordinary business is transacted, the speeches usual to such occasions being postponed till the public meeting at 7 P.M. Tea is provided for all visitors, governors, &c., and the hall in which the meetings take place is tastefully decorated with flowers and plants. These efforts to attract attendance at the hospital met with great success on March 26th, there being a satisfactory number at the Governors' meeting, and a large attendance at the public meeting, while a good many visitors took the opportunity afforded them of visiting the wards.

Alderman Strong took the chair at the Governors' meeting, at which Sir George Livesey was elected as President for the current year.

Sir George Livesey presided over the evening meeting, and in the course of his speech said he regretted to see from the report that a sum of £4,000 had been transferred from the Endowment Fund in order to provide for the ordinary expenditure of the year. Sir George went on to emphasise the fact that the experience of their hospital proved that alcohol was not necessary in cases of sickness.

Mr. E. Stafford Howard, who followed the chairman, said that the last year had been one of great prosperity for the hospital, the number of in-patients being larger than it has ever been before. During the 29 years in which the hospital had been in existence they had only administered alcohol in 56 cases.

Dr. Addinsell then said he wished to bring to the attention of the meeting the word efficiency, and congratulated the board on the improvement in the status of the junior staff and in the casualty department, which were now thoroughly efficient. The same, however, could not be said of all the departments. The meeting was then occupying a room which ought to be occupied by 20 beds; this increase would bring the establishment up to 100 beds, when they could have a medical teaching school, which was a very desirable factor; the out-patients' department was also manifestly inadequate. The point upon which the speaker laid the most stress was the necessity for the establishment of special departments for the study of the treatment of various diseases placed under the charge of special men. These were days of hospital reform and of hospital specialism, and no general physician or surgeon was able to keep pace with the enormous amount of special work being done. He understood that the hospital aimed at attaining the same degree of efficiency as that of the smaller metropolitan hospitals, but they would never obtain this as long as they did not adopt the departmental system. It was the quality of the work done and not the quantity which manifested the prosperity of the hospital, and the reputation of the hospital was entirely dependent upon the character of the individuals who constituted the staff and the quality of the work they turned out. If they wished to prove the principle that disease could be as successfully treated without alcohol as with, then they must put themselves in line with the other hospitals, and if they failed to do this they must fall behind in the race and their principle must suffer. If they really desired to see their principle popularised, and to do their best for the sick poor committed to their charge, then they would hearken to, even if they did not welcome, these criticisms and do their utmost to remedy these deficiencies which called loudly for reform.

Alderman Strong, Chairman of the Board of Management, assured the meeting that the board welcomed any criticism, and would always carefully consider any suggestions made with a view to promoting the success of the institution, but many of the points referred to by Dr. Addinsell would involve great additions and alterations, and therefore required a good deal of consideration. The board was, however, now as always ready to consider any well-matured suggestion that any of the staff might submit, especially those that made for the greater efficiency of the hospital.

### ST. MARY'S HOSPITAL.

Quite recently St. Mary's Hospital was honoured by a visit from its President, H.R.H. the Prince of Wales, who was accompanied by the Princess. Nearly every ward was visited, and their Royal Highnesses expressed themselves as very pleased with their visit, which they promised to repeat. This event was alluded to by the Chairman, Colonel Bird, when presiding at the annual meeting on Thursday, the 26th ult., and he proceeded further to say: "The Prince was, however, much struck with the darkness of the wards, and remarked how much healthier and nicer for the patients it would be to have the electric light. He was informed that the improvement would have been made long ago but for the great expense, the hospital being very poor and the cost of an electric light installation very heavy. He was told that the board had long been aware of the defect, and that attempts, with little or no good result, had been made to improve the gas lighting. His Royal Highness then requested that the board's attention should be specially drawn to the matter." The board, Colonel Bird added, had accordingly again considered the question, and, in deference to the wishes of the President, had definitely decided to light the hospital by electricity, and had appointed a committee to prepare a scheme and estimate for their consideration. The committee had not yet reported, therefore he could not say what the expense would be, though there was reason to think it would be about £2,500. This was a large outlay to have to face, but the board felt that the improvement ought not longer to be delayed. The committee was now sitting, and he hoped the installation would be complete before next winter.

### The Clarence Memorial Wing.

Substantial progress, said the Chairman, had been made with the Clarence Memorial wing, and the third floor was in course of construction. The handsome façade fronting Praed Street, with the legend at either corner: "£10,000 required to complete and furnish," arrested the attention of every passer-by, and the hope with which, sixteen years ago, the Praed Street extension was begun seemed likely to be realised, viz, that by bringing the hospital prominently into public notice a much larger measure of public support would be secured than had hitherto been possible. The Clarence Wing would add enormously to the importance of the hospital. A very large sum—at least £20,000—would be required for furnishing, and the Governors must set to work and show what they could do to raise it. There was still a great deal of building to be done, but he hoped the roof would be on by the summer.

### A Graceful Act.

The endowment of beds was a point he wished particularly to impress upon the subscribers and donors, as well as the public. There were now 40 endowed beds, and he could think of no more graceful act, in memory of a departed friend or relative, than to perpetuate his memory in this way. It was, however, of no use to suppose that £1,000 was sufficient for the purpose; the cost for maintenance had gone up to £96 per occupied bed, owing to the abnormal conditions under which the hospital must work until the buildings were complete, and the interest on £1,000 was not at all sufficient to support a bed. Still, it was very valuable and greatly appreciated. The chairman concluded by expressing the thanks of the board and the members of the medical and surgical staff; no one appreciated their work more than he did, nor realised more keenly how impossible it would be to carry on the business of the hospital without their frequent help. Two severe losses had been sustained in the retirement of Mr. Edmund Owen, senior surgeon, and Mr. Malcolm Morris, surgeon for diseases of the skin, on completion of the limit of service—20 years—prescribed in the laws of the hospital. The distinguished abilities and self-sacrificing devotion of these gentlemen to the interests of the hospital had done much to win for it the position which it occupied among the great hospitals of London to-day. In recognition of their services, both gentlemen had been elected to the consulting staff. Mr. Malcolm Morris, in addition to his work as a member of the staff, had, throughout his connection with the hospital, taken a prominent part in its administration as a member of the Board of Management, and in order to ensure the continuance of his assistance and counsel, which had in the past been productive of so much good to the institution, Mr. Morris had been elected a vice-president; this gave him a permanent seat on the board.

### ROYAL SEA-BATHING HOSPITAL, MARGATE.

The non-inclusion of the Royal Sea-Bathing Hospital in the list of hospitals receiving awards from King Edward's Fund was alluded to by several speakers at the annual court on Monday, March 23rd. Mr. F. C. Norton, in seconding the adoption of the report and accounts, said this was the first question that arrested attention. He was sorry to say it was a very severe shock to all the directors. The hospital was founded a hundred years ago for the benefit of the poor of London, and was placed at Margate because it was indispensable that Londoners should obtain sea air, and sea-bathing, as well as the other benefits which arose from the pure breezes of the North Sea. It had been thought that this hospital was fully entitled to share in the King's Fund; and the application was sent in in the hope and full expectation of its doing so. The answer received was however as follows:—"The committee do not deny the value of the services which your hospital renders to the population of London. The situation of your hospital is, however, so far beyond the radius within which the inspection of hospitals by our visitors is confined, that my committee have decided that your institution cannot be considered to come within the scope of the operations of this Fund." The directors, Mr. Norton continued, had no idea that there was any limit of distance, and they confidently believed that 19 out of every 20 subscribers to the Fund were equally ignorant. The disappointment was the more keenly felt, because it had compelled the directors to put up the charges, a course to which they felt great reluctance. This was, however, the only alternative, and last year the charges were raised for adults to 12s., and for children to 8s. per week. They would be very glad of financial help, which would enable them to reduce the terms, particularly in the case of the children, for while 8s. a week did not by any means represent the cost to the hospital, it did represent a very heavy drain upon a poor family. He asked subscribers to take note of the fact that no assistance was received from King Edward's Fund, and pointed out that if they desired to assist this hospital, it was necessary to subscribe directly to it, and not, as many supposed they were doing, to subscribe indirectly through the Fund. He thought the Council of the Fund should make known to the public what their limits of distance were. He was very glad to say that the generous promise of £500 made last year had been fulfilled; it was conditional upon an equal sum being raised by a certain date; £39 over the amount had been raised, and the same "well-wisher" had repeated the promise, allowing the balance to go towards the next amount. Owing to the very small sum received in legacies, the income in 1902 fell short of the expenditure by £1,113. The finances being low, the expenditure on improvements had been kept down as much as possible. An enlargement of the present kitchen was, however, a pressing necessity, and additional wards were required, especially for men, many of whom had to be kept waiting four or five weeks before they could be admitted. Representations had lately been made to the effect that there ought to be a ward for infants under six years of age. It was impossible to take them into the ordinary wards, and the directors hoped some day to have two wards for infants, with a separate staff of sisters and nurses.

The Chairman, Mr. Michael Biddulph, said he wished to add a word to what Mr. Norton had said about the grant from the King's Fund. It seemed very hard that this hospital was not included; it was not, he thought, in the position of a country hospital. It was a London hospital, with a London office, and he could not help thinking that if the committee were to look into the matter a little more they might see their way to giving a contribution. It was of no use having an office in London if the Hospital was not to share in the privileges of the Metropolitan hospitals, and he trusted that in future the decision might be reversed.

Another speaker said it would be most deplorable if patients had to be sent out before they were really well enough, for lack of funds. It was most earnestly to be hoped that King Edward's Fund would give a grant.

